# Evaluation of validity, reliability and ability to detect change for the Hand Eczema Severity Index (HECSI) and evaluation of HECSI‐75 and HECSI‐90 as within‐patient responder definitions

**DOI:** 10.1111/cod.14699

**Published:** 2024-10-13

**Authors:** Yasemin Topal Yüksel, Henrik Thoning, Lotte Seiding Larsen, Lucine Lehmann, Rob Arbuckle, Laura Grant, Tove Agner

**Affiliations:** ^1^ Department of Dermatology and Venereology Bispebjerg Hospital, University of Copenhagen Copenhagen Denmark; ^2^ LEO Pharma A/S Ballerup Denmark; ^3^ Adelphi Values Patient‐Centered Outcomes Cheshire UK

**Keywords:** Chronic Hand Eczema, Clinician‐Reported Outcome, delgocitinib cream, HECSI‐75, HECSI‐90, meaningful change, responder definition, validation

## Abstract

**Background:**

The Hand Eczema Severity Index (HECSI) is a Clinician‐Reported Outcome measure of the severity of hand eczema (HE).

**Objectives:**

This study aimed to evaluate the validity, reliability and ability to detect change of the HECSI, and the HECSI‐75 and HECSI‐90 as responder definitions.

**Methods:**

Analyses were performed using data from a sample of *n* = 258 patients with Chronic Hand Eczema (CHE) from a Phase 2b, randomised, double‐blind, vehicle‐controlled trial of delgocitinib cream, pooled across treatment groups. The measurement properties of the HECSI were assessed and the adequacy of the HECSI‐75 and HECSI‐90 as responder definitions was explored through cross‐tabulation.

**Results:**

Inter‐item correlations provided support for the scoring, whereby items are grouped by areas of the hand. HECSI demonstrated good test–retest reliability with intra‐class correlations >0.70. Construct validity was supported by a logical pattern of correlations with concurrent measures and significant differences in HECSI scores across severity groups (*p* < 0.001). HECSI was responsive with statistically significant improvements over time and with significant differences (*p* < 0.001) between improved and stable groups. Data provided support for both HECSI‐75 and HECSI‐90 as within‐patient responder definitions.

**Conclusions:**

HECSI has strong validity, reliability and ability to detect change as a measure of CHE severity. HECSI‐75 and HECSI‐90 are appropriate responder definitions.

## Graphical abstract

The Hand Eczema Severity Index (HECSI) is a Clinician‐Reported Outcome measure designed to assess the intensity and extent of six clinical hand eczema signs in five areas of the hand. Data from a clinical trial evaluating the efficacy of delgocitinib cream for treatment of Chronic Hand Eczema (CHE) were used to explore the psychometric properties of the HECSI and the appropriateness of the HECSI‐75 and HECSI‐90 as clinically meaningful within‐patient responder definitions. Results provide evidence that the HECSI is valid, reliable and sensitive to change in the context of use of CHE clinical trials and that the HECSI‐75 and HECSI‐90 are appropriate for defining clinically meaningful change.

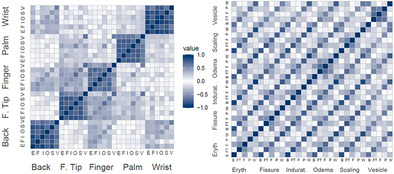

## 1 INTRODUCTION

Chronic Hand Eczema (CHE) is an inflammatory skin disease that is often multifactorial and has a high psychological, functional and occupational burden that warrants effective treatments.^1^, ^2^, ^3^ Hand eczema (HE) is referred to as Chronic Hand Eczema (CHE) when persisting for more than 3 months or relapsing twice or more per year.^3^ Morphology in the acute stage comprises erythema, oedema and vesicles, while dryness/scaling, papulation/induration and fissures are more common in the chronic stage. Commonly experienced symptoms include itch and pain.^4^ Assessment of eczema severity is important in clinical trials, epidemiological studies and clinical settings. Several objective scoring systems have successfully been used for severity assessment of CHE during recent decades.^5^, ^6^ The Hand Eczema Severity Index (HECSI) is designed to assess the intensity and extent of 6 clinical HE signs in five areas of the hand.^7^ It is the most widely used HE‐specific Clinician‐Reported Outcome (ClinRO) measure for assessing HE severity in clinical trials.^5^, ^8^

Previous work investigating the measurement properties of the HECSI has provided evidence of inter‐observer and intra‐observer reliability,^7^ ability to detect change over time,^9^ and estimates of thresholds for the smallest detectable change and minimally important differences.^9^, ^10^ However, dimensionality, construct validity and internal consistency have not previously been evaluated. Moreover, the performance of the measure when used in a clinical trial setting has not yet been investigated nor have within‐patient responder definitions in that context of use. Such evidence is of value to aid interpretation of HECSI scores and confirm its validity as a measure of CHE severity that can form an endpoint in clinical trials.

In the present study, we used data from a clinical trial evaluating the efficacy of delgocitinib cream as a treatment for CHE, to explore the validity, reliability and ability to detect change of the HECSI, and the appropriateness of HECSI‐75 and HECSI‐90 as clinically meaningful, within‐patient responder definitions.

## 2 METHODS

### 2.1 Study design and sample

The analyses described here were conducted using data from a Phase 2b dose‐ranging, double‐blind, multi‐centre, prospective, randomised, 5‐arm, vehicle‐controlled, parallel‐group trial evaluating the efficacy and safety of delgocitinib cream 1, 3, 8 and 20 mg/g compared to cream vehicle over a 16‐week treatment period in adult patients with CHE (Trial registration: NCT03683719). Full details of the Phase 2b study have been described elsewhere.^11^ Study activities were conducted in accordance with the Helsinki Declaration, and all participants provided written informed consent.

### 2.2 Participants

All patients who were randomised and exposed to treatment were included in the full analysis set (*n* = 258).

### 2.3 Measures

#### 2.3.1 Hand Eczema Severity Index

The HECSI is based on the assessment of signs (and does not include any patient‐reported items) and is a snapshot of the patient's HE at the time of examination.^7^, ^12^ To calculate a score on the HECSI, the patient's hand is divided into five areas (fingertips, fingers, palms, back of hands and wrists). For each area, the intensity of each of six clinical signs (erythema, induration/papulation, vesicles, fissuring, scaling and oedema) is graded as 0 (none/absent), 1 (mild), 2 (moderate) or 3 (severe). For each location (based on the total of both hands), the area is scored from 0 to 4 based on the extent of signs: 0 = 0%, 1 = 1%–25%, 2 = 26%–50%, 3 = 51%–75% and 4 = 76%–100%. The area score for each location is multiplied by the total sum of the intensity of each clinical feature. The total score ranges from 0 to 360 with higher scores indicating greater severity. The HECSI was assessed at screening, baseline and Weeks 1, 2, 4, 6, 8, 10, 12, 14 and 16.

#### 2.3.2 Convergent validity measures

The following clinician‐reported and patient‐reported outcomes (PROs) were included to (a) validate the HECSI in regard to convergent or discriminant validity (i.e., construct validity), or (b) define patients with stable CHE for test–retest reliability analysis.

The Investigator's Global Assessment for CHE (IGA‐CHE; adapted from Ruzicka et al.^13^) allows investigators to assess overall disease severity at one given time point and consists of a 5‐level severity scale (i.e., ‘0 = clear’, ‘1 = almost clear’, ‘2 = mild’, ‘3 = moderate’ and ‘4 = severe’).^14^ Each severity level on the scale is characterised in terms of the clinical characteristics of erythema, scaling, hyperkeratosis, vesiculation, oedema and fissures. The IGA‐CHE was assessed at screening, baseline and Weeks 1, 2, 4, 6, 8, 10, 12, 14 and 16.

Similarly, the Patient Global Assessment (PaGA) of disease severity is a single item 5‐level scale (0 = ‘clear’, 1 = ‘almost clear’, 2 = ‘mild’, 3 = ‘moderate’ and 4 = ‘severe’) which was completed by the patients at each visit to provide a patient‐reported global assessment of the HE severity. The PaGA was assessed at screening, baseline and Weeks 1, 2, 4, 6, 8, 10, 12, 14 and 16.

The Patient Global Impression of Change (PGI‐C) is a single item which asked patients to: ‘Please choose the response below that best describes the overall change in your HE since you started taking the study medication’. The five response options were: ‘much better’, ‘a little better’, ‘no change’, ‘a little worse’ and ‘much worse’. The PGI‐C was assessed at Weeks 4, 8 and 16.

The Dermatology Life Quality Index (DLQI)^15^ is a validated dermatology‐specific health‐related quality of life (HRQoL) questionnaire widely used as a trial endpoint in dermatological conditions to assess patients' perceptions of the impact of their skin disease on six domains of quality of life over the last week (symptoms and feelings, daily activities, leisure, work or school, personal relationships and treatment). Total score ranges from 0 to 30; higher scores indicate poorer HRQoL. The DLQI was assessed at screening, baseline and Weeks 1, 4, 8, 12 and 16.

The Quality of Life in Hand Eczema Questionnaire (QOLHEQ)^16^, ^17^ is another validated questionnaire used for assessment of quality of life in this study. However, in contrast to the DLQI, the QOLHEQ is a HE‐specific HRQoL questionnaire, that consists of 30 items, assessing disease‐related impairments over the last 7 days within four domains: (i) symptoms, (ii) emotions, (iii) functioning and (iv) treatment/prevention. The QOLHEQ was assessed at screening, baseline and Weeks 2, 6, 12 and 16.

### 2.4 Statistical analyses

The evaluation of validity, reliability and ability to detect change was conducted in accordance with European Medicines Agency (EMA) and Food and Drug Administration (FDA) standards and in particular the US FDA guidance on PROs and other clinical outcome assessments.^18^, ^19^, ^20^, ^21^, ^22^, ^23^, ^24^, ^25^, ^26^ The emphasis in a validation study such as this is to evaluate the magnitude of relationships between variables and the overall pattern of results. The following measurement properties were evaluated.

#### 2.4.1 Item score distributions

Distributions of ratings for each HECSI item were summarised at baseline, Weeks 8 and 16. Stacked bar charts were plotted for each area of the hand. Percentages of minimum and maximum ratings were also calculated to examine floor (a high percentage scoring the worst possible score [360]) and ceiling (a high percentage scoring the best possible score [0]) effects to ensure the rating scale is appropriate and can capture changes over time.

#### 2.4.2 Inter‐item correlations

Inter‐item correlations were examined to provide insights regarding the relationships among the HECSI items. A correlation matrix presenting correlations between each pair of items was produced using Week 4 data.

#### 2.4.3 Internal consistency and test–retest reliability

Internal consistency was assessed by calculating Cronbach's alpha for each area of the hand at Week 4. Cronbach's alpha coefficient ≥0.70 was considered evidence of acceptable internal consistency.^27^ Test–retest reliability was evaluated by examining the consistency of HECSI scores within a sample defined as having ‘stable’ CHE based on the stability of their scores on other measures between Weeks 12 and 16 and Weeks 14 and 16.^28^, ^29^ These time points were chosen as it was expected more patients would have stable CHE towards the end of the trial. Data were included even if assessors changed across time points. Stability was defined based on:

- no change on the IGA‐CHE between Weeks 14 and 16
- no change on the IGA‐CHE between Weeks 12 and 16
- <0.5 standard deviation (SD) change on the DLQI symptom domain between Weeks 12 and 16.
- <0.5 SD change on the QOLHEQ symptoms domain between Weeks 12 and 16

A modified intra‐class correlation coefficient (ICC) based on a multiple measurement, absolute agreement, two‐way mixed effects model was used to investigate test–retest reliability.^29^ ICC values were interpreted as follows: <0.50 indicates poor reliability; 0.50–0.75, moderate reliability; 0.75–0.90, good reliability; and >0.90 excellent reliability.^28^ A Pearson's correlation coefficient was also calculated as a sensitivity analysis.

#### 2.4.4 Construct validity: Convergent validity

Convergent validity of the HECSI scores was evaluated by examining correlations with scores of the IGA‐CHE, PaGA and DLQI at Week 4 and the QOLHEQ at Week 6 (the QOLHEQ was not assessed at Week 4). Convergent validity was considered to be met if there was a logical pattern of correlations, with scales measuring closely related concepts correlating more strongly than scales measuring concepts expected to be weakly related.^30^

#### 2.4.5 Construct validity: Known‐groups comparisons

The known‐groups method was used to evaluate differences in HECSI scores among groups of patients expected to differ in severity. Known‐groups were assessed using data from Week 8 (PaGA, IGA‐CHE and DLQI), apart from the analysis where known‐groups were defined using the QOLHEQ, which used data from Week 6. Full details of the known‐groups method are described in Supporting Information: Appendix 1.

#### 2.4.6 Ability to detect change over time

To evaluate ability to detect change over time, changes in HECSI scores between baseline and Week 16 were compared among groups defined as ‘improved’, ‘stable’ and ‘worsened’ based on changes in scores on: IGA‐CHE, PaGA, PGI‐C, DLQI (symptoms and feelings domain) and QOLHEQ (symptom domain). Within‐group effect sizes (ESs) (mean change score divided by the SD of the score at baseline) were calculated to evaluate the magnitude of changes in scores within each group.^31^

Between‐groups ESs were calculated using Hedge's *g* compared to the reference group.^32^ The following cut‐offs were used to interpret the magnitude of each ES: small (ES = 0.20), moderate (ES = 0.50) and large (ES = 0.80).^33^ Between‐groups ESs were calculated (using the same formula as in the known‐groups ES) for consecutive pairs of change groups. One‐way ANOVA was used to evaluate differences in change scores between each group.

#### 2.4.7 Responder definition: HECSI‐75 and HECSI‐90

As a measure of the treatment effect in skin diseases, a percentage reduction in severity score is often used and a trial endpoint/responder definition. In this study, HECSI‐75 and HECSI‐90 corresponding to 75% and 90% reduction in HECSI score from baseline, respectively, were assessed using cross‐tabulations to understand how meaningful each is as a within‐patient responder definition. The proportion of patients defined as within‐patient responders on the IGA‐CHE and PaGA were crossed with responders on the HECSI‐75 and HECSI‐90. Those achieving an IGA‐CHE score of 0 (clear) or 1 (almost clear) with at least a two‐level improvement were considered IGA‐CHE responders.^11^ PaGA responders were those achieving a PaGA score of 0 (clear) when classified at baseline as 1 (almost clear) or 2 (mild), or a PaGA score of 0 (clear) or 1 (almost clear) when classified at baseline as 3 (moderate) or 4 (severe).

#### 2.4.8 Treatment group differences

Differences in the proportions achieving HECSI‐75 and ‐90 were compared between treatment groups, with risk difference calculated using Mantel–Haenszel weighting taking stratification factors (baseline IGA‐CHE [mild, moderate and severe] and region [Europe and North America]) into account, with *p* values derived from a Cochran–Mantel–Haenszel test. Data collected after premature discontinuation of investigational medicinal product (IMP) or initiation of rescue medication were imputed as non‐responders. Any other missing data were also imputed as non‐responders.

## 3 RESULTS

### 3.1 Sample characteristics

Key demographic and clinical characteristics of the trial sample are provided in Table 1.

**TABLE 1:** Participant characteristics at baseline.

Characteristic	All randomised (*N* = 258)
Age	
Median (Q1–Q3)	48.0 (33.0–58.0)
Gender, *n* (%)	
Female	158 (61.2%)
Male	100 (38.8%)
Ethnicity, *n* (%)	
Hispanic or Latino	9 (3.5%)
Not Hispanic or Latino	249 (96.5%)
Race, *n* (%)	
White/Caucasian	254 (98.4%)
Asian	3 (1.2%)
Other (unspecified)	1 (0.4%)
Region, *n* (%)	
Europe	237 (91.9%)
North America	21 (8.1%)
Fitzpatrick skin type	
Type I	17 (6.6%)
Type II	145 (56.2%)
Type III	83 (32.2%)
Type IV	13 (5.0%)
CHE subtype (primary diagnosis)	
Atopic hand eczema	97 (37.6%)
Irritant contact dermatitis	80 (31.0%)
Hyperkeratotic hand eczema	46 (17.8%)
Acute recurrent vesicular hand eczema	20 (7.8%)
Allergic contact dermatitis	15 (5.8%)
Smoking history	
Smoker	79 (30.6%)
Ex‐smoker	70 (27.1%)
Non‐smoker	109 (42.2%)
IGA‐CHE severity score	
Clear	0 (0.0%)
Almost clear	0 (0.0%)
Mild	61 (23.6%)
Moderate	145 (56.2%)
Severe	52 (20.2%)
HECSI score	
Median (range)	44.5 (5–296)

Abbreviations: HECSI, Hand Eczema Severity Index; IGA‐CHE, Investigator's Global Assessment for Chronic Hand Eczema.

### 3.2 Item score distributions

All possible ratings were used for all items (i.e. ratings of 0 [none/absent], 1 [mild], 2 [moderate] or 3 [severe]), even at baseline. However, the severe option was rarely used across items; even at baseline, the highest proportion of patients in a severe category was 13.6% (finger scaling). For most items, the proportion of patients in the severe category was 0%–5%. As expected (based on improvements due to treatment), for all items, high proportions of patients had the severity of specific signs rated ‘none’/‘absent’ at Weeks 8 and 16, and ratings were grouped towards the lower end of the scale. A stacked bar chart presenting the Week 8 data is provided in Figure S1.

### 3.3 Inter‐item correlations

The tables of inter‐item correlations (Tables S1 and S2) are provided in Supporting Information. Correlations were highest between the items assessing signs in the same area of the hand. For example, the six items assessing signs for the fingertips all correlated with each other >0.50. This pattern of correlations provides support for the HECSI scoring, which includes domain scores made up of each area of the hand and a total score. For all parts of the hand, vesicles correlated less strongly with the other signs in a particular area, but the items assessing vesicles often correlated fairly strongly with the items assessing vesicles in other areas, for example, fingertip and finger vesicles (*r* = 0.72).

**FIGURE 1:**
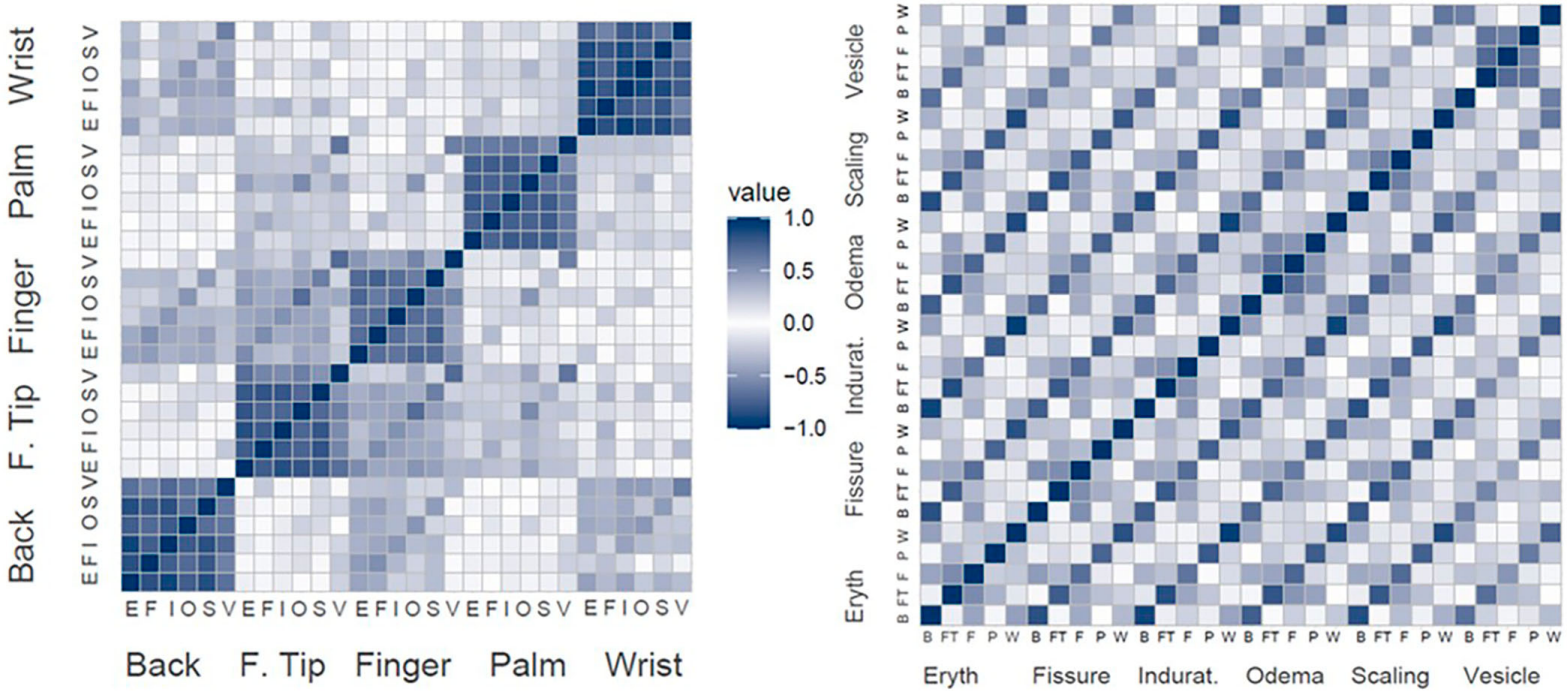
Correlation matrix heat map for the HECSI items at Week 4. Figure Key: E = erythema, F = Fissures, I = Induration, O = Oedema, S = Scaling, V = Vesicles, B = Back of the hand, FT = Fingertip, F = Finger, P = Palm, W = Wrist joint. HECSI, Hand Eczema Severity Index.

A correlation matrix heat map at Week 4 was also produced to help assess the inter‐item correlations (Figure 1). The heat map on the left shows the correlations between areas of the hand and the heat map on the right shows the correlations grouped by CHE signs. The clustering of high correlations (darker shading along the diagonal) when assessing correlations within each area of the hand provides support for grouping intensity items into the areas of the hand as in the current scoring (whereas there is no obvious pattern in the heat map on the right).

### 3.4 Internal consistency and test–retest reliability

Cronbach's alpha for the HECSI was assessed individually for each area of the hand due to the domain structure of the instrument (it is only appropriate to assess Cronbach's alpha on unidimensional scores).^34^ Cronbach's alpha for all areas of the hand scores were high (range: 0.86–0.89) and well above the a priori threshold of >0.70, indicating good internal consistency (Table S3).

The HECSI score demonstrated ‘good’ test–retest reliability (ICC = 0.77–0.83) when patients were defined as stable based on the DLQI and IGA‐CHE between Weeks 12 and 16; and ‘excellent’ test–retest reliability (ICC = 0.91) when patients were defined as stable based on the IGA‐CHE between Weeks 14 and 16. When the stable sample was defined based on minimal change on the QOLHEQ symptoms domain the results (ICC = 0.74) were slightly below the threshold of 0.75 for ‘good’ test–retest reliability (Table 2).

**TABLE 2:** Test–retest reliability analysis of HECSI between Weeks 12 and 16 and between Weeks 14 and 16.

Anchor for defining stability of CHE severity	Time point	*n* ^a^	ICC^b^ (95% CI)	Pearson's correlation coefficient
No change on the IGA‐CHE	Weeks 12–16	112	0.83 (0.77–0.88)	0.83
	Weeks 14–16	133	0.91 (0.87–0.93)	0.91
<0.5 SD change on QOLHEQ (symptoms domain)	Weeks 12–16	122	0.74 (0.65–0.81)	0.74
<0.5 SD change on DLQI (symptoms subscale)	Weeks 12–16	97	0.77 (0.67–0.84)	0.77

*Note*: ICC values were interpreted as follows: <0.50 indicates poor reliability; 0.50–0.75, moderate reliability; 0.75–0.90, good reliability; and >0.90 excellent reliability.^28^

Abbreviations: CI, confidence interval; DLQI, Dermatology Life Quality Index; HECSI, Hand Eczema Severity Index; ICC, intra‐class correlation; IGA‐CHE, Investigator's Global Assessment for Chronic Hand Eczema; QOLHEQ, Quality of Life in Hand Eczema Questionnaire; SD, standard deviation.

^a^ *n* represents the number of patients who are stable with regards to the anchor measure.

^b^ The ICC based on a single measurement, absolute agreement, two‐way mixed effects model was used.

### 3.5 Convergent validity

Correlations were examined between the HECSI scores and the IGA‐CHE, PaGA and DLQI (symptoms subscale), at Week 4 and with the QOLHEQ (symptom domain) at Week 6 (Table 3). Correlations ranged from 0.36 to 0.71, supporting convergent validity. A logical pattern of correlations was found, with HECSI correlating more strongly (0.71) with the IGA‐CHE, completed at the same visit by the same clinician, compared to the PROs (correlation range: 0.36–0.40). Known‐groups validity results also supported construct validity and are provided in Table S3.

**TABLE 3:** Convergent validity: Correlation of the HECSI with concurrent measures.

	IGA‐CHE at Week 4 (polyserial)	DLQI symptoms and feelings subscale at Week 4 (Pearson)	PaGA at Week 4 (polyserial)	QOLHEQ—Symptom domain at Week 6^a^ (Pearson)
HECSI	0.71 (*n* = 240)	0.36 (*n* = 239)	0.40 (*n* = 239)	0.39 (*n* = 225)

*Note*: Correlations were expected to be around 0.30–0.50 between measures of similar constructs.

Abbreviations: DLQI, Dermatology Life Quality Index; HECSI, Hand Eczema Severity Index; IGA‐CHE, Investigator's Global Assessment for Chronic Hand Eczema; PaGA, Patient Global Assessment; QOLHEQ, Quality of Life in Hand Eczema Questionnaire.

^a^ QOLHEQ was not collected at Week 4, hence the mix of weeks used.

### 3.6 Ability to detect change

Changes in HECSI scores between baseline and Week 16 were compared among the ‘improved’, ‘stable’ and ‘worsened’ groups defined using other trial endpoints as external anchors (Table 4). For all comparisons, the number of patients who worsened was extremely low and thus not focused on for interpretation here. For all anchors, there was strong evidence that the HECSI is responsive to improvements in CHE signs and symptoms over time, with consistently large ES improvements in the ‘improved’ groups, compared to moderate‐large ES in the ‘stable’ group. Statistically significant differences were found between the change groups, and mostly moderate or large ES between groups.

**TABLE 4:** Ability of the HECSI to detect change between Baseline and Week 16.

Grouping variable	*n*	Mean change score (SD)	Median change score (Min–Max)	Within‐groups effect size^a^	Between‐groups effect size^b^	Between‐groups *p* value^c^
IGA‐CHE						
≥1 level improvement	147	−48.59 (38.36)	−41.00 (−243.0 to 2.0)	−1.17		
Change score = 0	52	−10.25 (22.08)	−9.00 (−72.0 to 69.0)	−0.34	−0.99	
≥1 level worsening	6	11.00 (28.76)	11.00 (−34.0 to 47.0)	0.75	−0.73	<0.001
PaGA						
≥1 level improvement	134	−49.47 (39.83)	−41.00 (−243.0 to 2.0)	−1.14		
Change score = 0	57	−14.93 (25.41)	−11.00 (−64.0 to 69.0)	−0.53	−0.88	
≥1 level worsening	13	−6.08 (22.71)	−4.00 (−46.0 to 28.0)	−0.20	−0.31	<0.001
PGI‐C						
‘A little better’ and ‘Much better’	100	−50.39 (41.34)	−42.50 (−243.0 to 2.0)	−1.10		
‘No change’	20	−12.15 (28.78)	−7.00 (−70.0 to 47.0)	−0.58	−0.89	
‘A little worse’ and ‘Much worse’	6	6.17 (36.40)	−7.50 (−23.0 to 69.0)	0.29	−0.87	<0.001
QOLHEQ (symptom domain)						
≥0.5 SD improvement	147	−44.97 (40.50)	−36.00 (−243.0 to 47.0)	−1.04		
<0.5 SD change	44	−19.09 (22.04)	−19.50 (−64.0 to 33.0)	−0.73	−0.65	
≥0.5 SD worsening	13	−8.38 (40.81)	−4.00 (−79.0 to 69.0)	−0.30	−0.40	<0.001
DLQI (symptoms and feelings subscale)						
≥0.5 SD improvement	144	−45.87 (40.32)	−38.50 (−243.0–33.0)	−1.06		
<0.5 SD change	39	−19.56 (26.55)	−19.00 (−84.0–47.0)	−0.76	−0.65	
≥0.5 SD worsening	21	−9.10 (27.99)	−9.00 (−46.0–69.0)	−0.34	−0.40	<0.001

*Note*: The following cut‐offs were used to interpret the magnitude of each ES: small change (ES = 0.20), moderate change (ES = 0.50) and large change (ES = 0.80).^33^

Abbreviations: DLQI, Dermatology Life Quality Index; ES, effect size; HECSI, Hand Eczema Severity Index; IGA‐CHE, Investigator's Global Assessment for Chronic Hand Eczema; PaGA, Patient Global Assessment; PGI‐C, Patient Global Impression of Change; QOLHEQ, Quality of Life in Hand Eczema Questionnaire; SD, standard deviation.

^a^ Mean change score divided by the SD of the score at baseline.

^b^ Between‐group effect size to evaluate the magnitude of differences between groups. Calculated using Hedge's *g* as the difference between the two groups. Hedge's *g* is calculated as the difference in means divided by the pooled standard deviation. Each value in this column relates to the difference between the group in that row and the row above.

^c^ *F* test from a one‐way ANOVA.

### 3.7 Interpretation of score changes

The HECSI‐75 and HECSI‐90 were evaluated as possible responder definitions for defining within‐patient meaningful change by performing cross‐tabulations comparing patients achieving HECSI‐75 and HECSI‐90 against patients meeting responder definition thresholds for the anchor measures (Table 5). The proportion of responders on the IGA‐CHE and PaGA classified as responders on the HECSI‐75 was very high (96.6% and 90.4%, respectively).

**TABLE 5:** Cross‐tabulations of responders and non‐responders at Week 16 for the HECSI‐75 and HECSI‐90 compared against responder definitions for anchor scores.

	HECSI‐75		HECSI‐90	
	Responder	Non‐responder	Responder	Non‐responder
IGA‐CHE				
Responder, *N* (%)^a^	56 (96.6%)	2 (3.4%)	49 (84.5%)	9 (15.5%)
Non‐responder, *N* (%)	53 (36.1%)	94 (63.9%)	13 (8.8%)	134 (91.42%)
PaGA				
Responder, *N* (%)^b^	47 (90.4%)	5 (9.6%)	36 (69.2%)	16 (30.8%)
Non‐responder, *N* (%)	61 (40.1%)	91 (59.9%)	26 (17.1%)	126 (82.9%)

Abbreviations: HECSI, Hand Eczema Severity Index; IGA‐CHE, Investigator's Global Assessment for Chronic Hand Eczema; PaGA, Patient Global Assessment.

^a^ IGA‐CHE responder: an IGA‐CHE score of 0 (clear) or 1 (almost clear) with at least a two‐level improvement.

^b^ PaGA responder: a PaGA score of 0 (clear) when classified at baseline as 1 (almost clear) or 2 (mild), or a PaGA score of 0 (clear) or 1 (almost clear) when classified at baseline as 3 (moderate) or 4 (severe).

Additionally, the number of non‐responders on the IGA‐CHE and PaGA classified as responders by HECSI‐75 was sizeable (36.1% and 40.1%, respectively) compared to the small proportions of responders on the IGA‐CHE and PaGA who were classified as non‐responders on the HECSI‐75 (3.4% and 9.6%, respectively). Using the HECSI‐90, fewer responders on the IGA‐CHE and PaGA were classified as responders on the HECSI‐90 (84.5% and 69.2%, respectively) but importantly, fewer non‐responders on IGA‐CHE and PaGA were classified as responders by the HECSI‐90 (8.8% and 17.1%, respectively). However, it is also true that more responders on the IGA‐CHE and PGA were classified as non‐responders by the HECSI‐90 (15.5% and 30.8%, respectively).

Proportions of patients achieving HECSI‐75 and HECSI‐90 up to Week 16 stratified by treatment group are presented in Figure S2. When assessing HECSI‐75, there was a significantly higher proportion of responders observed in the 8 mg/g (57.7%; difference to vehicle: 31.2% [13.7%–48.8%], *p* = 0.001) and 20 mg/g (52.8%; difference to vehicle: 26.8% [8.9%–44.7%], *p* = 0.006) groups compared with the vehicle group (26.0%) after 16 weeks of treatment. When assessing HECSI‐90, there was a significantly higher proportion of responders observed in the 8 mg/g (40.4%; difference to vehicle: 30.1% [15.0%–45.2%], *p* = 0.000) and 20 mg/g (32.1%; difference to vehicle: 22.5% [7.3%–37.6%], *p* = 0.006) groups compared with the vehicle group (10.0%) after 16 weeks of treatment.

## 4 DISCUSSION

In this study, we provide evidence that the HECSI has strong validity and reliability and is sensitive to change in the context of use in clinical trials, and that HECSI‐75 and HECSI‐90 are clinically meaningful within‐patient responder definitions. At the item and total score levels of HECSI, distributions of scores were skewed towards the lower end of the scale and the 0 (‘none’/‘absent’) response was most often selected, which should be considered when interpreting the other validation results, particularly clinically important change thresholds. In part to address this issue, Oosterhaven et al. have proposed the following thresholds to aid interpretation of the HECSI total score: clear, 0; almost clear, 1–16; moderate, 17–37; severe, 38–116; and very severe, ≥117.^35^ The inter‐item correlations support the grouping of HECSI items into the areas of the hand prior to forming a total score (as in the specified scoring). Internal consistency results for the HECSI clinical signs within each area of the hand were above the a priori threshold of 0.70, suggesting that each of the six clinical signs assesses a single underlying trait. Test–retest results were very strong, irrespective of the time points used or how stability of CHE was defined.

Moderate to strong correlations with other measures assessing HE severity and HRQoL (IGA‐CHE, DLQI and QOLHEQ), provide evidence of convergent validity and that the HECSI is truly measuring CHE severity concepts that impact patients' HRQoL. As expected, correlations were highest with the IGA‐CHE, which is also a ClinRO measure of the severity of CHE signs. Slightly lower, but still moderate correlations with PRO measures of HRQoL, highlight the major impact of CHE on patients' functioning and wellbeing, which is consistent with the literature.^3^, ^36^ The correlation with the IGA‐CHE is higher than the correlations to PRO measures, which may be explained by the fact that the PRO measures assess signs and symptoms over a 7‐day recall period. Known‐groups comparisons showed that the HECSI is able to distinguish groups of patients who differ in CHE severity on other measures.

Importantly, the HECSI was shown to be sensitive to improvements in CHE severity, with large ESs within groups defined as ‘improved’ and between ‘improved’ and ‘stable’ groups.

In interventional studies using HECSI as the primary or secondary outcome, minimal important change values may be used to evaluate the effect of an intervention on HE severity. In the field of atopic dermatitis and psoriasis, responder definitions with 75% or 90% reductions in EASI or PASI, respectively, are widely used and accepted.^37^, ^38^, ^39^, ^40^ In this study, we found that HECSI‐75 and HECSI‐90 may be used as potential responder definitions. The data provide strong support for both HECSI‐75 and HECSI‐90 as responder definitions that can be used as endpoints for evaluating treatment response in clinical trials. The HECSI‐90 is considered more conservative as a threshold than the HECSI‐75 because fewer patients are classified as responders.

The IGA‐CHE assessment included in this study has also recently been validated,^14^ albeit a slightly updated version and using data from a later Phase 3 study, rather than the Phase 2b data used here. Both measures have demonstrated similarly strong reliability, construct validity and ability to detect change over time. Arguably these measures complement each other well, with the IGA‐CHE providing a global, single‐item assessment of CHE severity that can be quickly performed, whereas the HECSI provides a more detailed assessment incorporating specific assessments of all important CHE signs rated for each area of the hand, which are then combined to form a summary score.

### 4.1 Limitations

A limitation of this study is that the analyses were conducted in patients with mild to severe CHE at study entry and thus did not include patients who were ‘almost clear’. Thus, the sample may not be fully representative of the general CHE population. However, this was mitigated for by using later time points in the trial for all analyses, by which point some patients had improved to ‘clear’ or ‘almost clear’ due to treatment. Similarly, not all CHE subtypes were represented as there were no subjects with a primary diagnosis of protein contact dermatitis/contact urticaria. Some test–retest analyses were conducted at time points 4 weeks apart (i.e., Weeks 12 and 16). While these time points were selected as the points in the trial where patients were likely to have most stable disease, it should be acknowledged that 4 weeks is a relatively long period over which to expect stability.

### 4.2 Conclusion

In conclusion, our findings show that HECSI has good validity, reliability and ability to detect change over time, and therefore is fit‐for‐purpose as a ClinRO assessment of HE severity that is patient‐relevant. With respect to responder definitions, HECSI‐75 and HECSI‐90 are appropriate for defining clinically meaningful change.

## AUTHOR CONTRIBUTIONS

**Yasemin Topal Yüksel:** Writing – review and editing; supervision; methodology. **Henrik Thoning:** Writing – review and editing; supervision; methodology. **Lotte Seiding Larsen:** Writing – review and editing; conceptualization; supervision; methodology. **Lucine Lehmann:** Writing – review and editing; methodology; supervision. **Rob Arbuckle:** Writing – original draft; conceptualization; methodology; validation; supervision. **Laura Grant:** Writing – review and editing; conceptualization; methodology; validation; supervision; formal analysis. **Tove Agner:** Writing – review and editing; methodology; supervision.

## CONFLICT OF INTEREST STATEMENT

YTY has given lectures or participated in clinical studies for Pfizer, Karo Pharma, Pierre Fabre, LEO Pharma A/S and AbbVie. RA and LG are employees of Adelphi Values, a health outcomes consultancy contracted by LEO Pharma A/S to conduct the study. LHT and LL are employees of LEO Pharma A/S. LSL was an employee of LEO Pharma A/S at the time of performing the research and preparing the manuscript and is now an employee of H. Lundbeck A/S. TA has given lectures, participated in clinical studies, or been on advisory boards for Sanofi, LEO Pharma A/S, Pfizer, Eli Lilly, Galderma and AbbVie.

## Supporting information


**Data S1.** Supporting Information.

## Data Availability

The data that support the findings of this study are available from the corresponding author upon reasonable request.
