# Supplementary material for: Evaluation of validity, reliability and ability to detect change for the Hand Eczema Severity Index (HECSI) and evaluation of HECSI‐75 and HECSI‐90 as within‐patient responder definitions
Source: Contact Dermatitis. 2024 Oct 13;92(1):51–60. doi: 10.1111/cod.14699 (PMC11669562; doi:10.1111/cod.14699)
Supplement: Supplementary file 1 — Data S1. Supporting Information. [file COD-92-51-s001.docx]

**Title: Evaluation of validity, reliability and ability to detect change for the Hand Eczema Severity Index (HECSI) and evaluation of HECSI-75 and HECSI-90 as within-patient responder definitions**

**Authors:** Yasemin Topal Yüksel,^1^ Henrik Thoning^2^, Lotte Seiding Larsen^2^, Lucine Lehmann^2^, Rob Arbuckle^3^, Laura Grant^3^, and Tove Agner^1^

^1^Department of Dermatology, Bispebjerg University Hospital, Copenhagen, Denmark, ^2^LEO Pharma A/S, Ballerup, Denmark; ^3^Adelphi Values Patient-Centered Outcomes, Cheshire, UK.

**Corresponding author:** Rob Arbuckle, Adelphi Values, Grimshaw Ln, Bollington, Cheshire, SK10 5JB, UK; Tel: +44 (0)7720 880884; Email: [rob.arbuckle@adelphivalues.com](mailto:rob.arbuckle@adelphivalues.com)

## Table of contents:

Page 2-5 Supporting Tables 1-3

Page 6-7 Supporting Figures 1-2

Page 8-9 Appendix 1 – Known groups validity (methods and results)

Page 10 References

## Supporting Tables

| **Table S1. Inter-item correlations for HECSI at Week 4 in the primary analysis population (N=240) (items 1-15)** | | | | | | | | | | | | | | | |
| --- | --- | --- | --- | --- | --- | --- | --- | --- | --- | --- | --- | --- | --- | --- | --- |
| **Item** | **1** | **2** | **3** | **4** | **5** | **6** | **7** | **8** | **9** | **10** | **11** | **12** | **13** | **14** | **15** |
| Item 1 - fingertip: Erythema | 1.00 | . | . | . | . | . | . | . | . | . | . | . | . | . | . |
| Item 2 - fingertip: Fissures | 0.79 | 1.00 | . | . | . | . | . | . | . | . | . | . | . | . | . |
| Item 3 - fingertip: Infiltration/papulation | 0.85 | 0.80 | 1.00 | . | . | . | . | . | . | . | . | . | . | . | . |
| Item 4 - fingertip: Oedema | 0.82 | 0.74 | 0.71 | 1.00 | . | . | . | . | . | . | . | . | . | . | . |
| Item 5 - fingertip: Scaling | 0.80 | 0.79 | 0.80 | 0.74 | 1.00 | . | . | . | . | . | . | . | . | . | . |
| Item 6 - fingertip: Vesicles | 0.69 | 0.56 | 0.56 | 0.61 | 0.51 | 1.00 | . | . | . | . | . | . | . | . | . |
| Item 7 - finger: Erythema | 0.55 | 0.33 | 0.29 | 0.43 | 0.35 | 0.25 | 1.00 | . | . | . | . | . | . | . | . |
| Item 8 - finger: Fissures | 0.40 | 0.55 | 0.39 | 0.42 | 0.44 | 0.21 | 0.68 | 1.00 | . | . | . | . | . | . | . |
| Item 9 - finger: Infiltration/papulation | 0.40 | 0.44 | 0.52 | 0.44 | 0.41 | 0.21 | 0.66 | 0.69 | 1.00 | . | . | . | . | . | . |
| Item 10 - finger: Oedema | 0.49 | 0.39 | 0.43 | 0.70 | 0.38 | 0.39 | 0.73 | 0.62 | 0.71 | 1.00 | . | . | . | . | . |
| Item 11 - finger: Scaling | 0.44 | 0.40 | 0.35 | 0.44 | 0.62 | 0.18 | 0.71 | 0.76 | 0.68 | 0.61 | 1.00 | . | . | . | . |
| Item 12 - finger: Vesicles | 0.35 | 0.25 | 0.20 | 0.38 | 0.20 | 0.72 | 0.49 | 0.41 | 0.43 | 0.59 | 0.37 | 1.00 | . | . | . |
| Item 13 - palm: Erythema | 0.36 | 0.17 | 0.17 | 0.25 | 0.22 | 0.20 | 0.23 | 0.08 | 0.06 | 0.15 | 0.10 | 0.12 | 1.00 | . | . |
| Item 14 - palm: Fissures | 0.29 | 0.37 | 0.29 | 0.24 | 0.21 | 0.28 | 0.10 | 0.31 | 0.18 | 0.19 | 0.06 | 0.12 | 0.73 | 1.00 | . |
| Item 15 - palm: Infiltration/papulation | 0.26 | 0.191 | 0.28 | 0.21 | 0.22 | 0.27 | 0.02 | 0.04 | 0.22 | 0.19 | 0.05 | 0.13 | 0.78 | 0.75 | 1.00 |
| Item 16 - palm: Oedema | 0.42 | 0.34 | 0.35 | 0.54 | 0.29 | 0.41 | 0.18 | 0.10 | 0.19 | 0.49 | 0.07 | 0.24 | 0.79 | 0.75 | 0.77 |
| Item 17 - palm: Scaling | 0.28 | 0.27 | 0.26 | 0.26 | 0.37 | 0.17 | 0.10 | 0.14 | 0.15 | 0.18 | 0.27 | 0.07 | 0.76 | 0.77 | 0.79 |
| Item 18 - palm: Vesicles | 0.28 | 0.25 | 0.11 | 0.25 | 0.18 | 0.66 | 0.11 | 0.02 | 0.02 | 0.22 | 0.03 | 0.62 | 0.64 | 0.62 | 0.66 |
| Item 19 - back of hand: Erythema | 0.06 | -0.03 | -0.08 | 0.02 | 0.03 | -0.20 | 0.43 | 0.38 | 0.20 | 0.23 | 0.30 | -0.07 | 0.04 | -0.00 | -0.09 |
| Item 20 - back of hand: Fissures | -0.01 | -0.06 | -0.09 | -0.17 | 0.00 | -0.27 | 0.47 | 0.56 | 0.33 | 0.21 | 0.27 | -0.00 | -0.06 | 0.07 | -0.09 |
| Item 21 - back of hand: Infiltration/papulation | 0.11 | 0.04 | 0.08 | 0.15 | 0.08 | -0.19 | 0.36 | 0.41 | 0.50 | 0.29 | 0.33 | -0.09 | 0.03 | 0.06 | 0.08 |
| Item 22 - back of hand: Oedema | 0.04 | -0.01 | -0.08 | 0.22 | -0.20 | -0.00 | 0.38 | 0.38 | 0.33 | 0.50 | 0.20 | 0.18 | -0.02 | 0.10 | -0.06 |
| Item 23 - back of hand: Scaling | 0.08 | 0.03 | 0.03 | 0.05 | 0.18 | -0.19 | 0.40 | 0.45 | 0.34 | 0.21 | 0.46 | -0.03 | 0.06 | 0.08 | -0.01 |
| Item 24 - back of hand: Vesicles | 0.02 | -0.24 | -0.03 | -0.03 | -0.19 | -0.03 | 0.28 | 0.28 | 0.31 | 0.18 | 0.20 | 0.36 | 0.05 | -0.02 | 0.05 |
| Item 25 - wrist joint: Erythema | 0.11 | -0.09 | -0.04 | 0.12 | -0.10 | -0.04 | 0.26 | 0.09 | -0.06 | 0.20 | 0.09 | 0.05 | 0.30 | 0.20 | 0.15 |
| Item 26 - wrist joint: Fissures | -0.21 | -0.27 | -0.37 | -0.14 | -0.37 | -0.18 | 0.02 | -0.05 | -0.22 | -0.06 | -0.03 | -0.10 | 0.10 | 0.30 | 0.05 |
| Item 27 - wrist joint: Infiltration/papulation | 0.05 | -0.04 | -0.03 | 0.10 | -0.11 | -0.05 | 0.16 | 0.07 | 0.09 | 0.23 | 0.11 | 0.09 | 0.20 | 0.17 | 0.15 |
| Item 28 - wrist joint: Oedema | 0.01 | -0.26 | -0.09 | 0.17 | -0.31 | 0.01 | 0.06 | -0.03 | 0.01 | 0.26 | -0.05 | 0.11 | 0.27 | 0.19 | 0.19 |
| Item 29 - wrist joint: Scaling | -0.08 | -0.09 | -0.11 | 0.04 | -0.04 | -0.23 | 0.12 | 0.05 | -0.10 | 0.06 | 0.14 | 0.10 | 0.19 | 0.12 | 0.09 |
| Item 30 - wrist joint: Vesicles | 0.03 | -0.34 | -0.04 | 0.05 | -0.29 | -0.20 | 0.04 | -0.01 | -0.04 | 0.10 | -0.01 | -0.04 | 0.22 | 0.10 | 0.18 |
| **Key:**   \|  \| 0.8 - 0.89 \| \| --- \| --- \| \|  \| 0.7 -0.79 \| \|  \| 0.6 – 0.69 \| \|  \| 0.5 – 0.59 \| \|  \| 0.4 - 0.49 \| \|  \| 0.3 - 0.39 \| \|  \| <0.3 \| | | | | | | | | | | | | | | | |

| **Table S2. Inter-item correlations for HECSI at Week 4 in the primary analysis population (N=240) (items 16-30)** | | | | | | | | | | | | | | | |
| --- | --- | --- | --- | --- | --- | --- | --- | --- | --- | --- | --- | --- | --- | --- | --- |
| **Item** | **16** | **17** | **18** | **19** | **20** | **21** | **22** | **23** | **24** | **25** | **26** | **27** | **28** | **29** | **30** |
| Item 16 - palm: Oedema | 1.00 | . | . | . | . | . | . | . | . | . | . | . | . | . | . |
| Item 17 - palm: Scaling | 0.67 | 1.00 | . | . | . | . | . | . | . | . | . | . | . | . | . |
| Item 18 - palm: Vesicles | 0.63 | 0.53 | 1.00 | . | . | . | . | . | . | . | . | . | . | . | . |
| Item 19 – back of hand: Erythema | -0.06 | -0.05 | -0.11 | 1.00 | . | . | . | . | . | . | . | . | . | . | . |
| Item 20 - back of hand: Fissures | -0.09 | -0.11 | -0.24 | 0.86 | 1.00 | . | . | . | . | . | . | . | . | . | . |
| Item 21 - back of hand: Infiltration/papulation | 0.06 | 0.03 | -0.18 | 0.91 | 0.79 | 1.00 | . | . | . | . | . | . | . | . | . |
| Item 22 - back of hand: Oedema | 0.22 | -0.09 | 0.04 | 0.78 | 0.71 | 0.79 | 1.00 | . | . | . | . | . | . | . | . |
| Item 23 - back of hand: Scaling | -0.02 | 0.13 | -0.09 | 0.84 | 0.80 | 0.84 | 0.70 | 1.00 | . | . | . | . | . | . | . |
| Item 24 - back of hand: Vesicles | 0.00 | -0.06 | 0.07 | 0.68 | 0.61 | 0.76 | 0.64 | 0.63 | 1.00 | . | . | . | . | . | . |
| Item 25 - wrist joint: Erythema | 0.21 | 0.22 | 0.25 | 0.47 | 0.17 | 0.37 | 0.41 | 0.36 | 0.38 | 1.00 | . | . | . | . | . |
| Item 26 - wrist joint: Fissures | 0.12 | 0.22 | 0.31 | 0.28 | 0.37 | 0.14 | 0.38 | 0.35 | 0.38 | 0.89 | 1.00 | . | . | . | . |
| Item 27 - wrist joint: Infiltration/papulation | 0.22 | 0.22 | 0.25 | 0.42 | 0.21 | 0.47 | 0.41 | 0.40 | 0.42 | 0.93 | 0.81 | 1.00 | . | . | . |
| Item 28 - wrist joint: Oedema | 0.33 | 0.18 | 0.27 | 0.23 | -0.01 | 0.32 | 0.52 | 0.23 | 0.47 | 0.86 | 0.73 | 0.93 | 1.00 | . | . |
| Item 29 - wrist joint: Scaling | 0.05 | 0.18 | 0.26 | 0.38 | 0.16 | 0.26 | 0.24 | 0.50 | 0.37 | 0.88 | 0.82 | 0.88 | 0.80 | 1.00 | . |
| Item 30 - wrist joint: Vesicles | 0.21 | 0.08 | 0.17 | 0.32 | 0.03 | 0.27 | 0.235 | 0.20 | 0.66 | 0.74 | 0.57 | 0.82 | 0.76 | 0.63 | 1.00 |
| **Key:**   \|  \| 0.8 - 0.89 \| \| --- \| --- \| \|  \| 0.7 -0.79 \| \|  \| 0.6 – 0.69 \| \|  \| 0.5 – 0.59 \| \|  \| 0.4 - 0.49 \| \|  \| 0.3 - 0.39 \| \|  \| <0.3 \| | | | | | | | | | | | | | | | |

| **Table S3. Internal consistency reliability: Cronbach’s alpha for the HECSI clinical signs within each area of the hand at Week 4 (N=240)** | | | | | |
| --- | --- | --- | --- | --- | --- |
|  | **Fingertip** | **Fingers** | **Palm** | **Back of hand** | **Wrist joint** |
| Cronbach’s alpha | 0.89 | 0.86 | 0.89 | 0.87 | 0.88 |
| alpha value ≥0.70 considered indicative of good internal consistency reliability. | | | | | |

## Supporting Figures

**Figure S1. Item response distribution for the HECSI at Week 8**


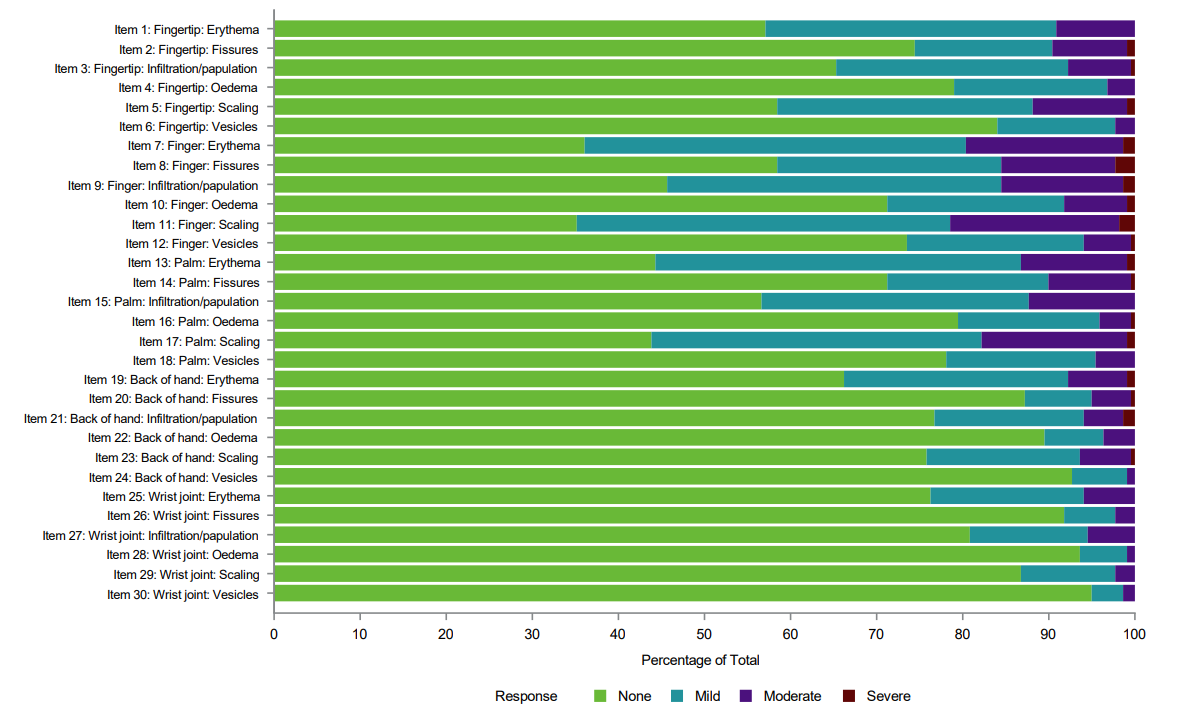


.

**Figure S2. Proportions of patients achieving HECSI-75 and HECSI-90 up to week 16, by treatment group for the mild to severe population. Proportion of responders achieving HECSI-75 (A) and HECSI-90 (B).**

**A: HECSI-75**

**
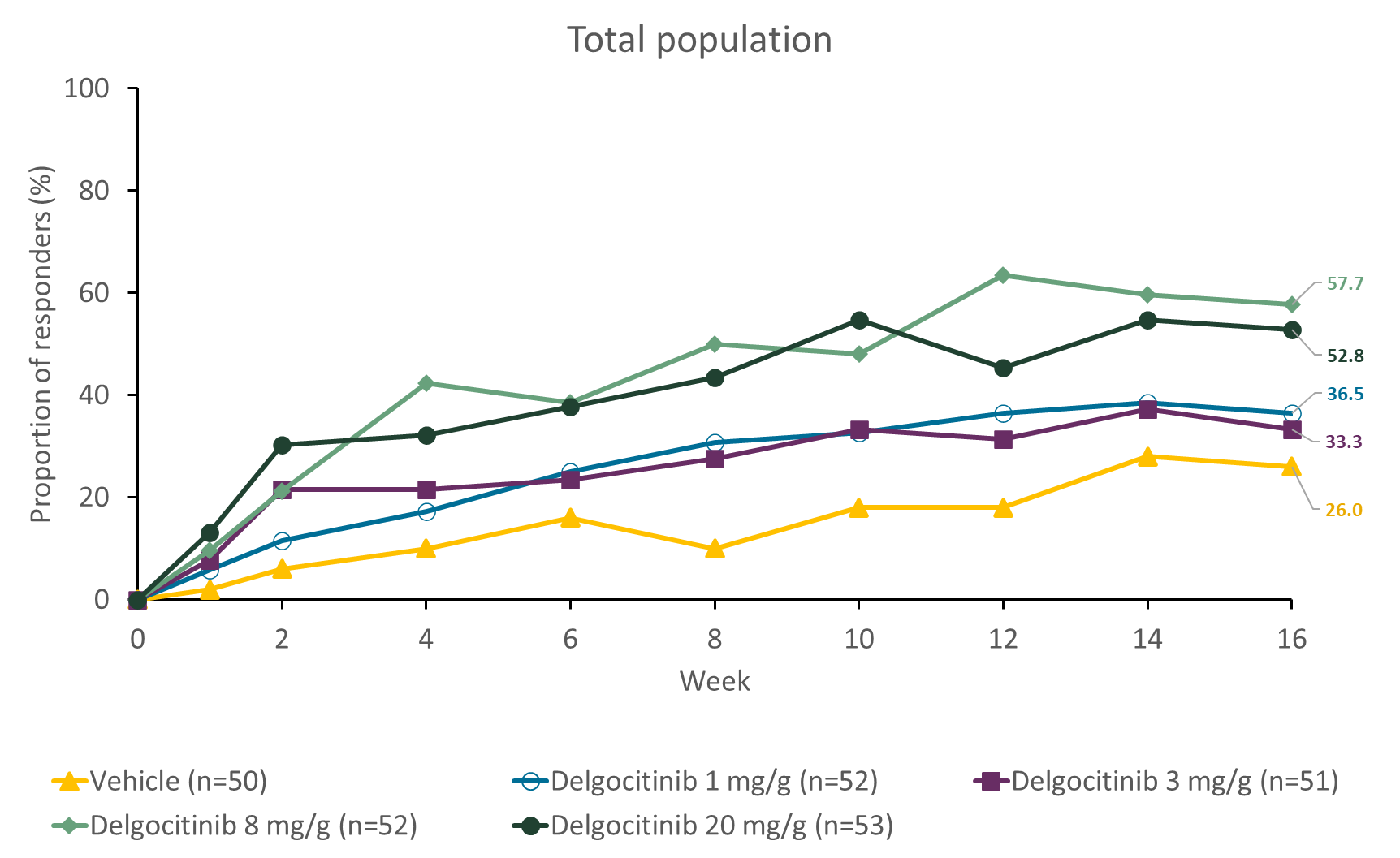
**

**B: HESCI-90**

**
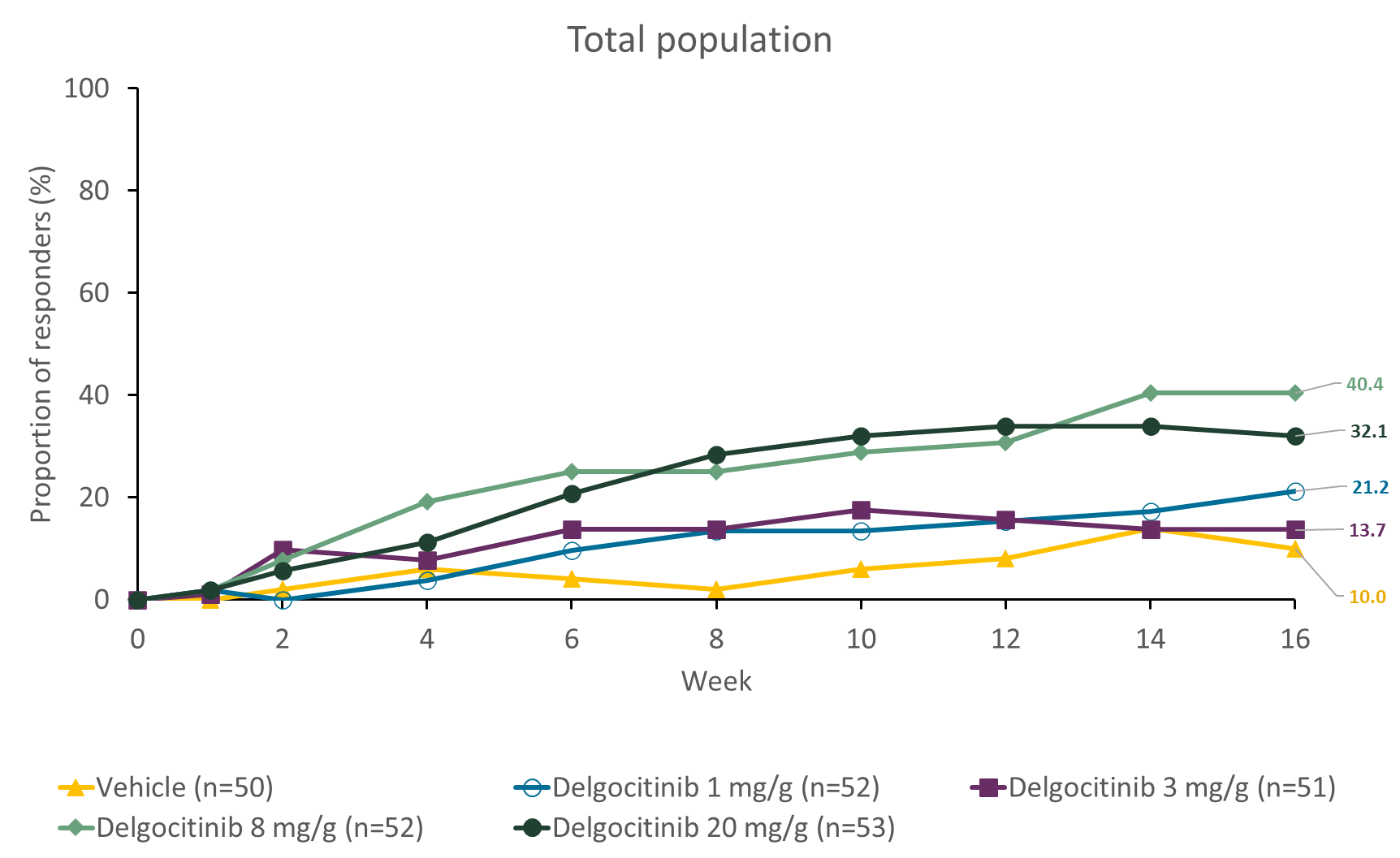
**

All outcomes are based on the full analysis set, p-values were derived from a Cochran-Mantel-Haenszel test stratified by baseline IGA-CHE [mild, moderate, and severe] and region [Europe and North America]. Data collected after premature discontinuation of investigational medicinal product (IMP) or initiation of rescue medication were imputed as non-responders. Any other missing data were also imputed as non-responders. CHE, Chronic Hand Eczema; HECSI, Hand Eczema Severity Index.

## Appendix 1. Known groups validity

*Known groups validity: methods*

Known groups validity involves examining differences in scores between groups expected to differ (i.e. due to being of differing levels of CHE severity).^1^ HECSI scores were compared between groups of patients who differed on variables hypothesized to influence the construct of interest. The following comparisons were made:

- PaGA: 0-1 (clear or almost clear), 2 (mild), 3 (moderate), 4 (severe)
- IGA-CHE: 0-1 (clear or almost clear), 2 (mild), 3 (moderate), 4 (severe)
- QOLHEQ item 6 (itch): <median score at Week 6, ≥ median score at Week 6
- DLQI item 1 (itchy, sore, painful, stinging): < median score at Week 8, ≥ median score at Week 8

Week 8 was chosen for this analysis due to the similarities between known groups and convergent validity, allowing for further confirmation of the results. The statistical significance (p≤0.05) of differences in scores between groups was also calculated using one-way Analysis of variance (ANOVA). Between groups effect sizes (ES) were calculated using Hedge’s g compared to the reference group. Hedge’s g is calculated as the difference in means divided by the pooled standard deviation. The pooled standard definition was used as the denominator when assessing the ES of a group difference and was calculated as follows:

$${SD}_{Pooled}=\sqrt{\frac{{(n_{1}-1)SD}_{1}^{2}+{(n_{2}-1)SD}_{2}^{2}}{n_{1}+n_{2}-2}}$$

The following cut-offs were used to interpret the magnitude of each effect size: small (ES = 0.20), moderate (ES = 0.50), and large (ES = 0.80).^2^

*Known groups validity: results*

When known groups were defined using the PaGA and IGA-CHE, the ‘severe’ and ‘moderate’ groups were merged because the sample sizes for the severe groups were less than 20. As displayed in Table S4, the expected pattern of worse scores for severity groups defined as more severe with monotonically increasing mean scores across groups was demonstrated for all known group comparisons, with statistically significant difference among the groups. Moreover, with just one exception all between-group effect sizes were moderate or large (ES=0.59-1.68), suggesting substantial differences between the groups. Thus, the known groups comparisons provide support for the construct validity of the HECSI.

| **Table S4. Known-groups validity analysis of the HECSI at Week 6 or Week 8** | | | | | |
| --- | --- | --- | --- | --- | --- |
| **Grouping Variable** | **n** | **Mean HECSI Score (SD)** | **Between groups effect size [1]** | **Linear trends p-value [2]** | **Pairwise tests p-value [3]** |
| **Week 8: IGA-CHE** | | | | | |
| 0-1 (Clear or almost clear) | 49 | 2.35 (1.96) |  | <0.0001 |  |
| 2 (mild) | 99 | 16.47 (12.00) | -1.43 |  | <0.0001 |
| 3-4 (moderate or severe) | 71 | 43.90 (32.10) | -1.68 |  | <0.0001 |
| **Week 8: PaGA** | | | | | |
| 0-1 (Clear or almost clear) | 44 | 6.91 (11.41) |  | <0.0001 |  |
| 2 (mild) | 100 | 16.97 (18.99) | -0.59 |  | 0.0001 |
| 3-4 (moderate or severe) | 74 | 38.32 (30.71) | -1.24 |  | <0.0001 |
| **Week 6: QOLHEQ Total score** | | | | | |
| < median score at Week 6 | 103 | 14.50 (17.28) |  | N/A |  |
| ≥ median score at Week 6 | 112 | 29.55 (29.86) | -0.61 |  | <0.0001 |
| **Week 6: QOLHEQ Item 6: itch** | | | | | |
| < median score at Week 6 | 68 | 15.04 (18.77) |  | N/A |  |
| ≥ median score at Week 6 | 147 | 25.71 (27.78) | -0.42 |  | <0.0001 |
| **Week 8: DLQI Total Score** | | | | | |
| < median score at Week 8 | 96 | 12.88 (16.15) |  | N/A |  |
| ≥ median score at Week 8 | 121 | 29.70 (29.19) | -0.69 |  | <0.0001 |
| **Week 8: DLQI Item 1: itchy, sore, painful, stinging** | | | | | |
| < median score at Week 8 | 25 | 5.76 (7.48) |  | N/A |  |
| ≥ median score at Week 8 | 192 | 24.41 (26.40) | -0.75 |  | <0.0001 |
| [1] Calculated using Hedge’s g, which is calculated as the difference in means between adjacent groups divided by the pooled standard deviation  [2] A General Linear model test of linear trends. If significant it suggests that the group differences are increasing or decreasing monotonically  [3] T-tests used for pairwise comparisons between sets of consecutive groups  The following cut-offs were used to interpret the magnitude of each effect size: small (ES = 0.20), moderate (ES = 0.50), and large (ES = 0.80) | | | | | |

## References

1. Fayers PM, Machin D. *Quality of life: the assessment, analysis and reporting of patient-reported outcomes*. John Wiley & Sons; 2015.

2. Cohen J. *Statistical Power Analysis for the Behavioral Sciences*. Taylor & Francis; 2013.
